# Tapping Performance of Professional and Amateur Darbuka Players

**DOI:** 10.3389/fpsyg.2022.861821

**Published:** 2022-06-30

**Authors:** Kazuaki Honda, Shinya Fujii

**Affiliations:** ^1^Graduate School of Media and Governance, Keio University, Fujisawa, Japan; ^2^NTT Communication Science Laboratories, NTT Corporation, Atsugi, Japan; ^3^Faculty of Environment and Information Studies, Keio University, Fujisawa, Japan

**Keywords:** darbuka players, motor skills, rhythm, tapping, coordination

## Abstract

Motor skills of professional musicians can be regarded as a model to investigate human skill acquisition after prolonged practice. Although rhythmic tapping skills of musicians such as drummers and pianists were investigated previously, the tapping performance of hand percussionists is still largely unknown. In this study, we investigated the tapping performance of professional and amateur darbuka players. Three tapping tasks were performed: single-, double-, and triple-finger tapping tasks. The participants were asked to tap as fast as possible for 12 s in the single-finger tapping task while they tapped as fast and alternate/even as possible in the double- and triple-finger tapping tasks. The tapping speed and variability of inter-tap interval (ITI) and tapping amplitude were assessed for each task. In the single-finger and triple-finger tapping tasks, there was no significant difference in the tapping speed between the professional and amateur darbuka players. In the double-finger tapping task, the tapping speed was significantly faster in the professional players than the amateur players. Interestingly, the professional players showed faster tapping speed in both familiar and unfamiliar patterns of finger coordination. The tapping speed of the double-finger tapping task was significantly correlated with the duration and the age of commencement of darbuka training. The professional players also showed less variability of ITI and tapping amplitude compared to the amateur players. These results suggest that prolonged practice of the hand percussion increases the performance stability and coordination speed of both familiar and unfamiliar patterns.

## Introduction

After prolonged practice, musicians show rapid, rhythmic motor performance. For example, an expert pianist plays 1,800 notes per minute with the hands to play the 6th Paganini-Etude by Franz Liszt (Münte et al., 2002). The winner of a contest to find the world’s fastest drummer plays about 1,200 beats per minute with the hands (Fujii et al., 2009; Fujii and Moritani, 2012a,b). Musicians are therefore considered an ideal population to investigate behavioral and brain changes in the hand-motor system after extensive practice (Münte et al., 2002; Schlaug, 2003). To assess the skilled manual performance, a fast rhythmic tapping task, which asks a participant to tap as fast as possible in dozens of seconds, has been used in many previous studies (Peters and Durding, 1978; Jäncke et al., 1997; Lutz et al., 2004; Aoki et al., 2005; Fujii and Oda, 2006; Rüber et al., 2015).

The previous studies have shown that the rhythmic tapping speed depends on at least three factors: First, the tapping speed depends on the digit to be used. Aoki et al. (2005) asked pianists and non-pianists to tap either with a thumb, an index, a middle, a ring, or a little finger as fast as possible by using the metacarpophalangeal joint movement. They showed that the tapping speed of an index or a middle finger was faster than that of a ring or a little finger in both pianists and non-pianists. Interestingly, there was no significant difference in the speeds of the index and middle fingers between the pianists and the non-pianists, while a difference was found in the speeds of the ring and little fingers between the groups. The results suggest that piano practice increases the tapping speed at the ring and little fingers but not at the index and middle fingers.

Second, the tapping speed depends on the degree of hand dominance. Many tapping studies have shown that the tapping speed of a preferred hand was faster than that of a non-preferred hand (Peters and Durding, 1978; Jäncke et al., 1997; Lutz et al., 2004; Fujii and Oda, 2006). Therefore, the degree of tapping-speed asymmetry has been used as a measure of hand dominance (Peters and Durding, 1978). Previous studies on musicians showed that the degree of tapping-speed asymmetry was reduced in drummers, keyboard, and string-instrument players compared with non-musicians (Amunts et al., 1997; Jäncke et al., 1997; Fujii and Oda, 2006). These studies suggest that musical practice reduces tapping-speed asymmetry between the hands.

Third, the tapping speed decreases when the finger and hand are coordinated. For example, when we tap at a maximum speed while coordinating two fingers alternatively, the tapping speed per finger is slower than that performed without finger coordination. The tapping speed in the double-finger tapping task is slower than that in the single-finger tapping task (Aoki et al., 2005). Another example is the difference between unimanual and bimanual tapping performances. When we coordinate two hands at maximum speed, the bimanual tapping speed is slower than the unimanual tapping speed (Fujii et al., 2010). Previous studies showed that musicians were faster and more stable than non-musicians when coordinating the movements of fingers and hands (Yamanishi et al., 1980; Verheul and Geuze, 2004; Aoki et al., 2005; Fujii et al., 2010).

Taken together, the tapping speed depends on the digit to be used, the hand dominance, and the way of manual coordination. Also, these factors are thought to interact with the degree of experience of musical practice. However, the instrument-specific effect on tapping performance remains unclear.

Recently, Matthews et al. (2016) compared the rhythmic tapping performance among drummers, pianists, singers, string players, and non-musicians. They found that the musicians clearly outperformed compared with non-musicians, yet the difference among the subgroups of musicians was unclear. Their finding suggests that there may be little or no instrument-specific effect on the finger tapping performance. On the contrary, the other studies suggest that the tapping performance may be different among subgroups of musicians. For example, Jäncke et al. (1997) showed the difference between keyboard- and string-instrument players: The keyboard-instrument players showed less tapping-speed asymmetry than the string-instrument players (Jäncke et al., 1997). This could be due to the fact that a keyboard-instrument player uses both left- and right-hand fingers to play a keyboard, while a string-instrument player uses left fingers more than the right to hold down the strings (Bangert and Schlaug, 2006). Nevertheless, the tapping skills of the other musical instrument players are still largely unknown.

**Figure 1:**
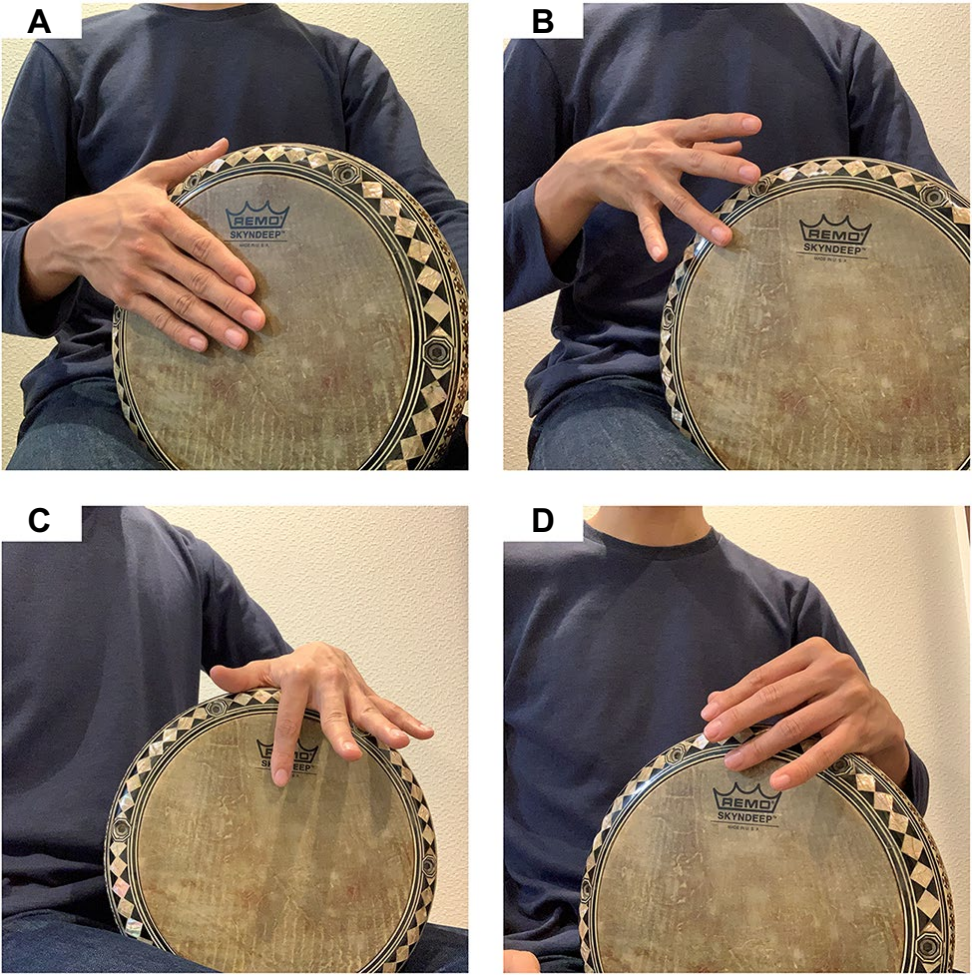
An example of playing a percussion “darbuka” by a right-handed musician. To play the darbuka, the players mainly make three sounds named “Doum” (a low-pitch sound), “Pa” (a slapping sound), and “Tek” (a high-pitch sound). The Doum and Pa sounds are made by striking the center of a drum head by a preferred hand **(A)**. The Tek sound is made by striking the edge of a drum head with a ring finger of a preferred hand **(B)**, or an index or a ring finger of a non-preferred hand **(C,D)**.

In this study, we focused on the tapping skills of hand percussionists who play the percussion called “darbuka,” a single-headed drum widely used throughout the Middle East (Figure 1A). Darbuka players are considered to be an interesting population to investigate the rhythmic manual skills because the players practice the finger and hand movements in a unique way (Karaol and Doğrusöz, 2014). To play the darbuka, the players mainly make three sounds named as (1) “Doum” (a low-pitch sound), (2) “Pa” (a slapping sound), and (3) “Tek” (a high-pitch sound). The Doum and Pa sounds are made by striking the center of a drum head with a preferred hand (Karaol and Doğrusöz, 2014). The Doum sound is made by keeping the palm and fingers straight with all fingers firmly closed to each other, while the Pa sound is made by keeping the hand shape like scooping water. The Tek sound is made by striking the edge of a drumhead with a ring finger of a preferred hand (Figure 1B) or an index or a ring finger of a non-preferred hand (Figures 1C,D).

The skills to play darbuka is unique in terms of which fingers to be used, coordination of fingers, and asymmetry of hands. First, darbuka players mainly use three fingers. For instance, the “split-finger” technique invented by Misirli Ahmet in the 1980s is well-known as a skill to perform the Tek sounds very quickly. The technique involves the sequential tapping movements of a ring finger of a preferred hand, the index and ring fingers of a non-preferred hand. Thus, not all fingers but specific fingers are contacted the drum surface during the practice of playing darbuka. Second, darbuka players show asymmetrical manual movements when they play the instrument. That is, darbuka players use the movements of the preferred hand more than that of the non-preferred hand because low-pitch sounds play an important role in the rhythm pattern of Middle Eastern music. Specifically, the players use the preferred hand more than the non-preferred hand to make the Doum and Pa sounds for playing rhythm patterns in Middle Eastern music.

We consider that darbuka players form a unique population for investigating the effect of musical practice on tapping skills. While keyboard players contact the keys with all their fingers to play the instrument, the darbuka players contact the drum surface with the specific fingers (i.e., a ring finger of a preferred hand and an index and ring fingers of a non-preferred hand). Drum-kit players mainly use a drumstick to hit the drum surface while the darbuka players contact with the fingers (Fujii and Oda, 2009; Eriksen et al., 2018). Therefore, we consider that darbuka players shed unique light on the instrument-specific effect on tapping performance after extensive practice. However, as far as we know, there has been no study investigating the tapping skills of darbuka players.

The purpose of this study was therefore to investigate the tapping skills of darbuka players. Specifically, we aimed to test the following hypotheses considering the previous tapping studies: First, we hypothesized that right-handed professional darbuka players would show faster tapping speed than amateur players when they tapped with the familiar fingers to play darbuka (i.e., left-index, left-ring, and right-ring fingers). Second, we hypothesized that professional and amateur darbuka players would show similar tapping-speed asymmetry because both professional and amateur darbuka players practice preferred hand more to play the rhythm patterns in Middle Eastern music. Third, we hypothesized that professional darbuka players would show faster tapping performance than amateur players when they tapped alternately/sequentially with the familiar fingers to play darbuka (left-index, left-ring, and right-ring fingers). To test these hypotheses, we compared the tapping speed of professional darbuka players and amateur controls. Additionally, we investigated the variability of inter-tap interval (ITI) and tapping amplitude to compare the performance stability of the professional and amateur players.

## Materials and Methods

### Participants

Japanese professional darbuka players (8 males, mean age = 37.25 years, standard deviation [SD] = 5.66, range = 30–50 years) and Japanese amateur darbuka players (8 males, mean age = 41.50 years, SD = 9.34, range = 28–55 years) participated in this study (see Table 1). The professional players had experiences of earning money from their musical performances and teaching students, whereas the amateur players did not have any of those experiences. The professional players started to play the darbuka earlier than the amateur players (professional players: mean age of commencement = 24.38 years, SD = 4.27, range = 21–34 years, amateur players: mean age of commencement = 35.63 years, SD = 10.80, range = 25–51 years; *t*(14) = 2.74, *p* = 0.023). The duration of darbuka training of professional players is longer than that of the amateur players (professional players: duration = 13.75 years, SD = 2.77, range = 8–17 years, amateur players: duration = 6.38 years, SD = 4.92, range = 4–18 years; *t*(14) = −3.69, *p* = 0.02). The handedness of participants was determined using the Edinburgh Handedness Inventory (Oldfield, 1971). All participants were right-handed (professional players: mean laterality quotient [LQ] = 92.37, SD = 10.54, range = 80–100, amateur players: mean LQ = 92.37, SD = 15.01, range = 60–100). The two groups were matched for sex, age and handedness. The experimental procedure was approved by the Ethics Committee of Keio University Shonan Fujisawa Campus (No. 161) and informed consent was obtained from all participants.

**Table 1:** Age, age of commencement, handedness score for both groups (SD and range in parentheses).

	Mean age (years)	Mean age of commencement (years)	Mean duration of darbuka training (years)	Mean laterality quotient
Professional darbuka players (*n* = 8)	37.25 (5.66, 30–50)	24.38 (4.27, 21–34)	13.75 (2.77, 8–17)	92.37 (10.54, 80–100)
Amateur darbuka players (*n* = 8)	41.50 (9.34, 28–55)	35.63 (10.80, 25–51)	6.38 (4.92, 4–18)	92.37 (15.01, 60–100)

All participants for both groups are male.

### Task

There were three tasks in this study: (1) single-finger, (2) double-finger, and (3) triple-finger tapping tasks. In the single-finger tapping task, there were four conditions: left-index (Li), left-ring (Lr), right-index (Ri), and right-ring (Rr) finger conditions. The Li, Lr, and Rr were familiar conditions and Ri was an unfamiliar condition. In the double-finger tapping task, there were six conditions denoted as Li–Lr, Ri–Rr, Li–Ri, Lr–Rr, Li–Rr and Lr–Ri. For example, the Li-Lr indicates the alternate coordination of the left-index and left-ring fingers. The Li–Lr, Lr–Rr and Li–Rr were familiar coordination for the right-handed darbuka players because they usually tap with right-ring, left-ring, and left-index fingers to play the instrument. On the other hand, the Ri–Rr, Li–Ri, and Lr–Ri were unfamiliar to the right-handed darbuka players. The participants were asked to coordinate the fingers as fast and alternate as possible and to start the tapping from the preferred right hand when they coordinated the left and right hands. They started from the index finger when they coordinated the index and ring fingers in a hand. In the triple-finger tapping task, there was only one condition denoted as Rr–Li–Lr (coordination of right-ring, left-index, and left-ring fingers), which is known as the split finger technique (Karaol and Doğrusöz, 2014). In this technique, the participants were asked to coordinate the right-ring, left-index, and left-ring fingers sequentially in this order. The participants were asked to tap as fast as possible in the single-finger tapping task while they were asked to tap as fast and alternate/even as possible in the double- and triple-finger tapping tasks for 12 s after a start call of an experimenter. Note that the participants in this study were asked to tap with the fingers but allowed to use upper-arm joints movements without any constraints as they usually play the instrument. This was because we aimed to investigate the natural performance seen in actual darbuka playing. For each of the conditions in each of the tasks, the participants performed three trials each. The participants were asked to have one-minute rest between the trials to prevent fatigue. The order of the task and conditions were randomized among the participants.

### Setup and Data Acquisition

The height and position of the drum chair were adjusted for each participant to be comfortable. The participants were asked to hold a darbuka (Aluminum die-cast model, Egygwhara) under the left upper arm. A microphone (M50, Earthworks) was located 50 cm apart from the center of the percussion head. The signals of the microphone were transformed from analog to digital at 192 kHz sampling frequency and 24-bit quantization by an audio interface (F8, ZOOM).

### Data Analysis and Statistics

**Figure 2:**
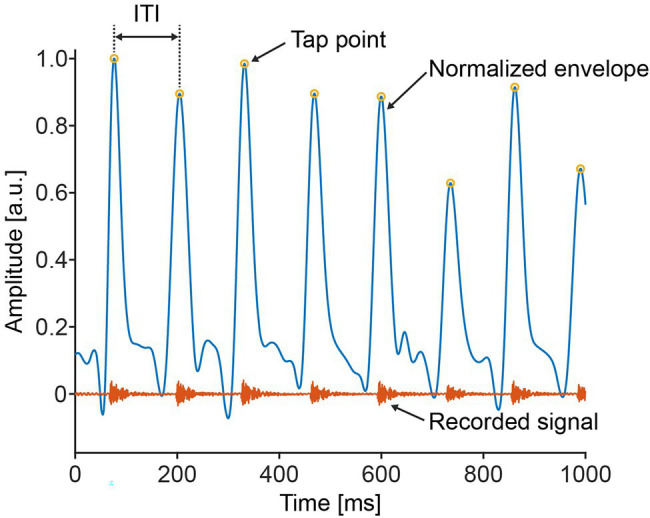
A typical example of the recorded sound signal. The envelope of the recorded signal was calculated and the amplitude was normalized by the maximum value. The “tap points” were detected from the envelope signal. The inter-tap intervals (ITI) were calculated as the interval between the two sequential tap points.

The tapping speed was assessed with the following steps. First, the audio data were rectified, and the envelope was calculated by using the Hilbert transform. The envelope signal was then normalized by the maximum value for each trial. From the normalized envelope signal, we detected a local maximum above 10% of the maximum value as the “tap points” (see Figure 2). The ITI in this study was defined as the interval between two sequential tap points.

We truncated the first and last parts of the 12-s tap-points data to eliminate the effects of start-up and final slowing and used the data during a middle 10-s period. We calculated ITIs from the 10-s tap-points data and removed outliers of ITI by using the median absolute deviation for each trial. We then pooled the ITI data from three trials for each condition and created linear mixed-effects models (LMMs) to test the hypotheses on tapping speed. We first entered Task (single/double/triple) and Group (professional/amateur) and their interaction as fixed effects in the LMM to test if the group and task differences affect the tapping speed. Participants and trials were entered as random effects in the LMMs to account for the inter-individual and inter-trial differences.

As for the ITI data from the single-finger tapping task, we used the LMM that had the fixed effects of Group (professional/amateur), Finger (ring/index), Hand (right/left) and their interactions. Specifically, we tested whether the professional players showed faster tapping speed with their familiar fingers by entering Group (professional/amateur) and Familiarity [familiar (Lr, Li, and Rr)/unfamiliar (Ri)] and their interaction as fixed effects in the LMM. Also, to test if the professional and amateur players showed similar tapping-speed asymmetry, we used the LMM that had Group and Hand and their interaction as the fixed effects.

As for the ITI data from the double-finger tapping task, we used the LMM that had the fixed effects of Group (professional/amateur) and Familiarity [familiar (Li-Lr, Lr-Rr and Li-Rr)/unfamiliar (Ri-Rr, Li-Ri, and Lr-Ri)] and their interaction to test if the professional players coordinated the familiar fingers faster than the amateur players.

To assess the variability of tapping performance, we calculated the coefficient of variance (CV) of ITI and CV of tapping amplitude. The CV of tapping amplitude was calculated from the 10-s tap-points data for each trial. The CV of ITI was calculated from the 10-s ITI data after the removal of outliers using the median absolute deviation for each trial. To test if the performance stability differed between the professional and amateur players, we used the LMMs that had Task (single/double/triple) and Group (professional/amateur) and their interaction as the fixed effects. In all the LMMs, participants and trials were entered as random effects to account for the inter-individual and inter-trial differences.

In one professional player, we recorded the signals at a 48 kHz sampling frequency and missed a double-finger tapping task (Rr–Li). We omitted the sound signals of an amateur player in a single-tapping task (Li) due to the too low signal-to-noise ratio to detect the taps. Because the tapping frequency is enough slower than the sampling frequency of the sound signal and the LMMs have the robustness for missing values, we conducted the LMMs including these participants.

To investigate if the tapping speed of an individual was related to the experience of musical training, we performed correlation analyses. We calculated the tapping frequency as the inverse of the median ITI for each trial. The tapping frequencies were then averaged using the data from the double-finger tapping task (see “Results” for the reason why the data from the double-finger tapping task were used). We tested if the age of commencement of darbuka training and the duration of darbuka training were correlated with the tapping frequency to investigate whether earlier commencement of training and longer duration of training lead to faster tapping performance (Jäncke et al., 1997; Bailey and Penhune, 2010; Kopiez et al., 2010; Rüber et al., 2015). We examined how linear and polynomial functions were able to explain the data variance in the correlations. We used Spearman’s rho correlation coefficients to assess the relationships.

The data processing was performed with MATLAB software (R2021b, Mathworks), and the statistical analyses were performed by R software. The LMMs were performed by using the “lmer” function in the “lme4” package for R software (Bates et al., 2015). Wald Chi-Square tests were used to reveal significant main effects and interactions in the LMMs. The statistical results were deemed as significant at the level of *p* < 0.05 for each of the statistical analyses. We calculated the partial eta squared ( $\eta_{p}^{2}$ ) values as the effect sizes. In the Supplementary Material, we described the details about all the LMMs we used for the analyses.

## Results

### Effects of Task and Group on Tapping Speed

**Figure 3:**
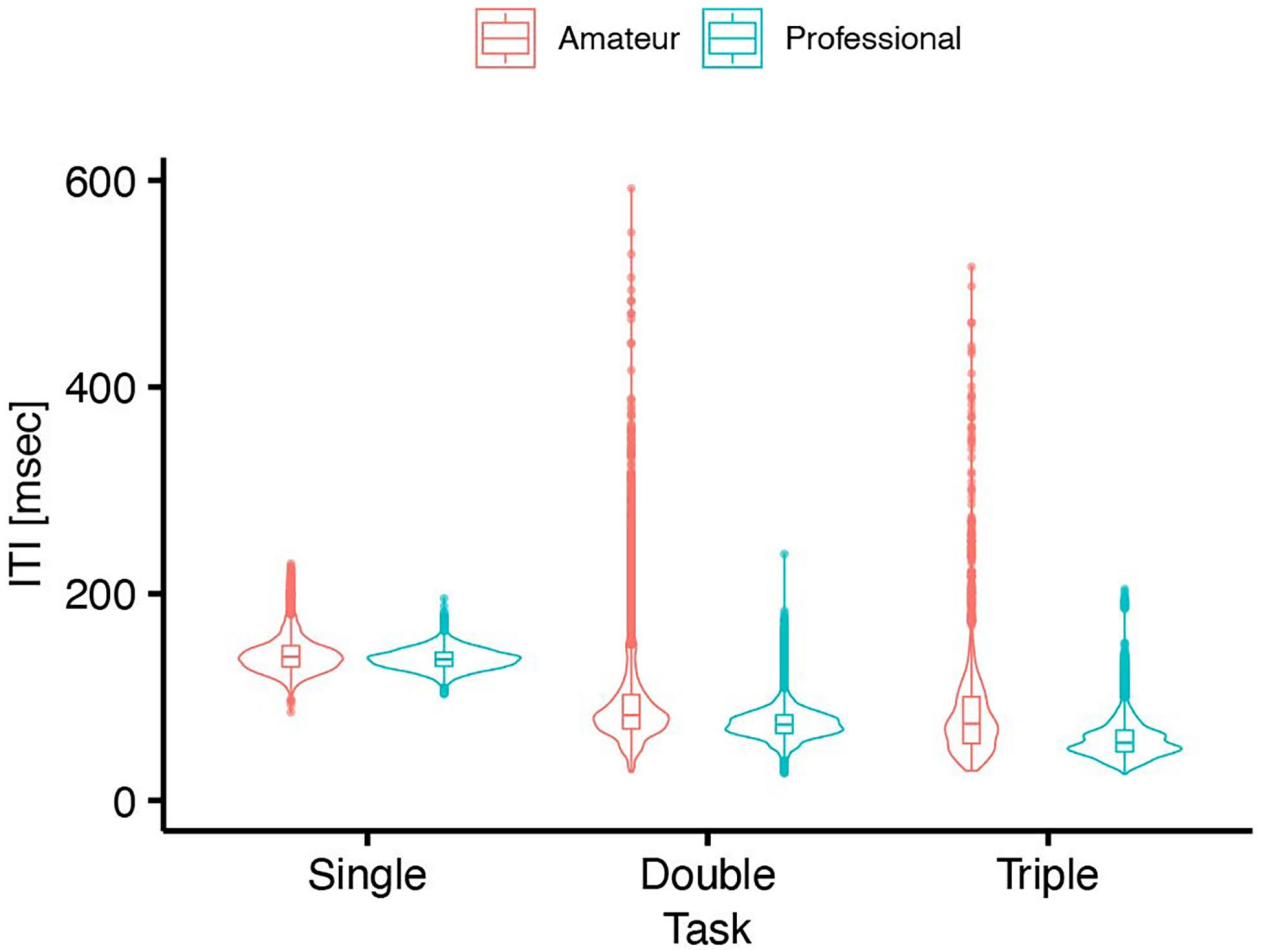
Inter-tap interval of professional and amateur darbuka players in the single-, double- and triple-finger tapping tasks (*n* = 8 for the professional group and *n* = 8 for the amateur group).

The LMM on ITI revealed a significant interaction between Task and Group ( $\chi^{2}$ (2) = 913.40, *p* < 0.01, $\eta_{p}^{2}$ = 0.019, Figure 3). The main effect of Task was significant ( $\chi^{2}$ (2) = 13390.90, *p* < 0.01, $\eta_{p}^{2}$ = 0.46) while the main effect of Group was not significant ( $\chi^{2}$ (1) = 0.72, *p* = 0.40). *Post hoc* LMM analyses entering Group as the fixed effect for each task showed the significant main effect of Group in the double-finger tapping task $(\chi^{2}$ (1) = 5.77, *p* = 0.016, $\eta_{p}^{2}$ = 0.29). On the other hand, the main effect of Group was not significant in the single-finger tapping task $(\chi^{2}$ (1) = 1.21, *p* = 0.27) nor in the triple-finger tapping task $(\chi^{2}$ (1) = 3.12, *p* = 0.077). Thus, the professional players performed faster than the amateur players only in the double-tapping task ( β = −0.022, *t* = −2.40, *95%* CI = −0.039– −0.0031). The detailed results on the LMMs were summarized in the Supplementary Material.

### Tapping Speed in Single-Finger Tapping Task

**Figure 4:**
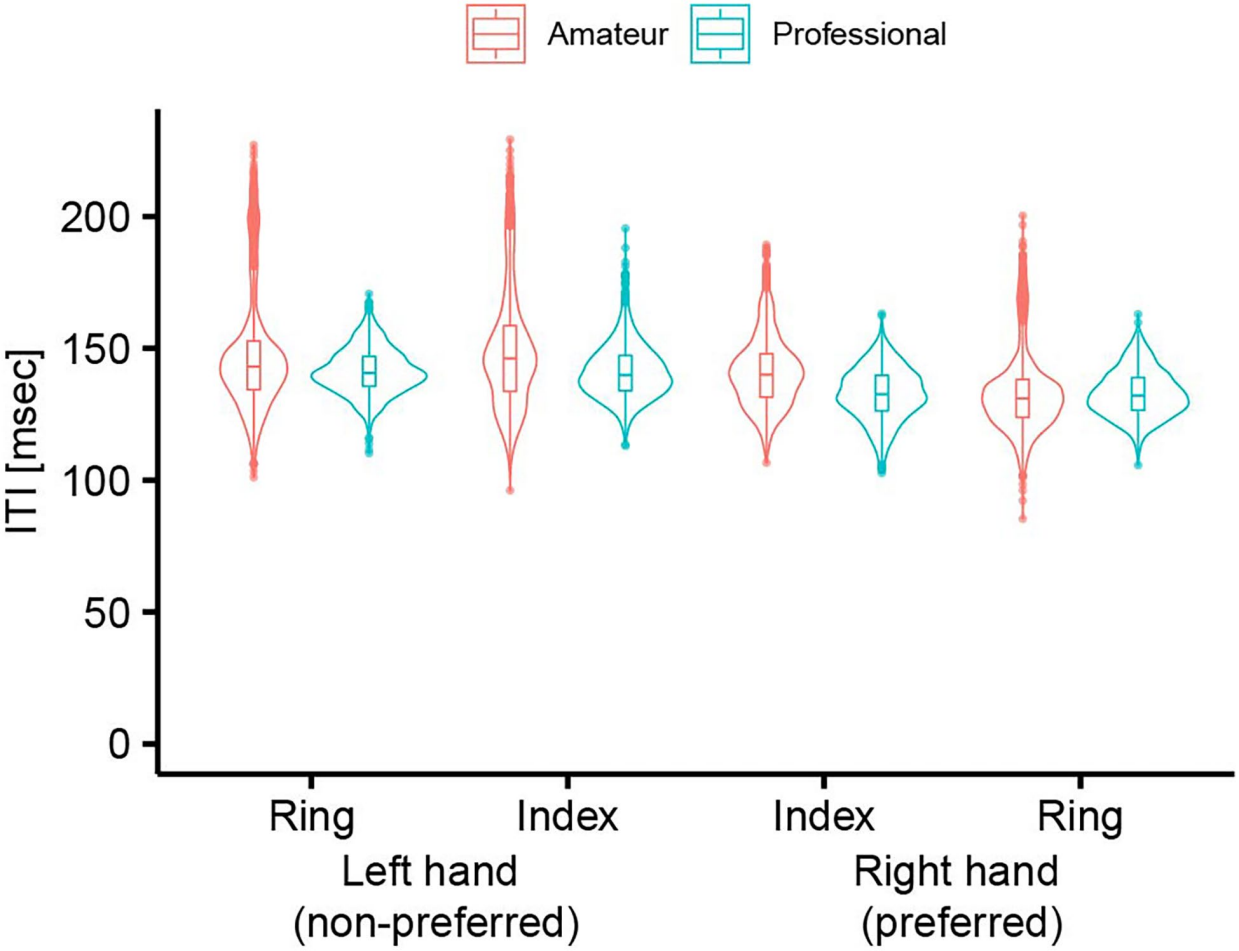
Inter-tap interval of professional and amateur darbuka players in the single-finger tapping task (*n* = 8 for the professional group and *n* = 8 for the amateur group).

The LMM on ITI revealed significant interaction among Group, Finger, and Hand in the single-finger tapping task [ $\chi^{2}$ (1) = 55.12, *p* < 0.01, $\eta_{p}^{2}$ = 0.0047, Figure 4]. The LMM also revealed a significant interaction between Group and Familiarity [ $\chi^{2}$ (1) = 15.07, *p* < 0.01, $\eta_{p}^{2}$ = 0.0013]. However, *post hoc* LMM analyses entering Group as the fixed effect for each familiarity showed no significant main effect of Group [familiar, $\chi^{2}$ (1) = 0.96, *p* = 0.33; unfamiliar, $\chi^{2}$ (1) = 2.66, *p* = 0.10, respectively].

We found a significant interaction between Group and Hand in the single-finger tapping task [ $\chi^{2}$ (1) = 189.27, *p* < 0.01, $\eta_{p}^{2}$ = 0.02]. *Post hoc* LMM analyses entering Hand as the fixed effect for each group showed that there was a significant main effect of Hand in both of the groups [professional, $\chi^{2}$ (1) = 1598.16, *p* < 0.01, $\eta_{p}^{2}$ = 0.20; amateur, $\chi^{2}$ (1) = 1706.53, *p* < 0.01, $\eta_{p}^{2}$ = 0.24, respectively]. Nevertheless, the significant interaction showed that the professional players had less degree of tapping-speed asymmetry between hands ( β = 0.0085, *t* = 39.98, 95% CI = 0.0081–0.0089) compared to the amateur players ( β = 0.014, *t* = 41.31, 95% CI = 0.013–0.014).

### Tapping Speed in Double-Finger Tapping Task

**Figure 5:**
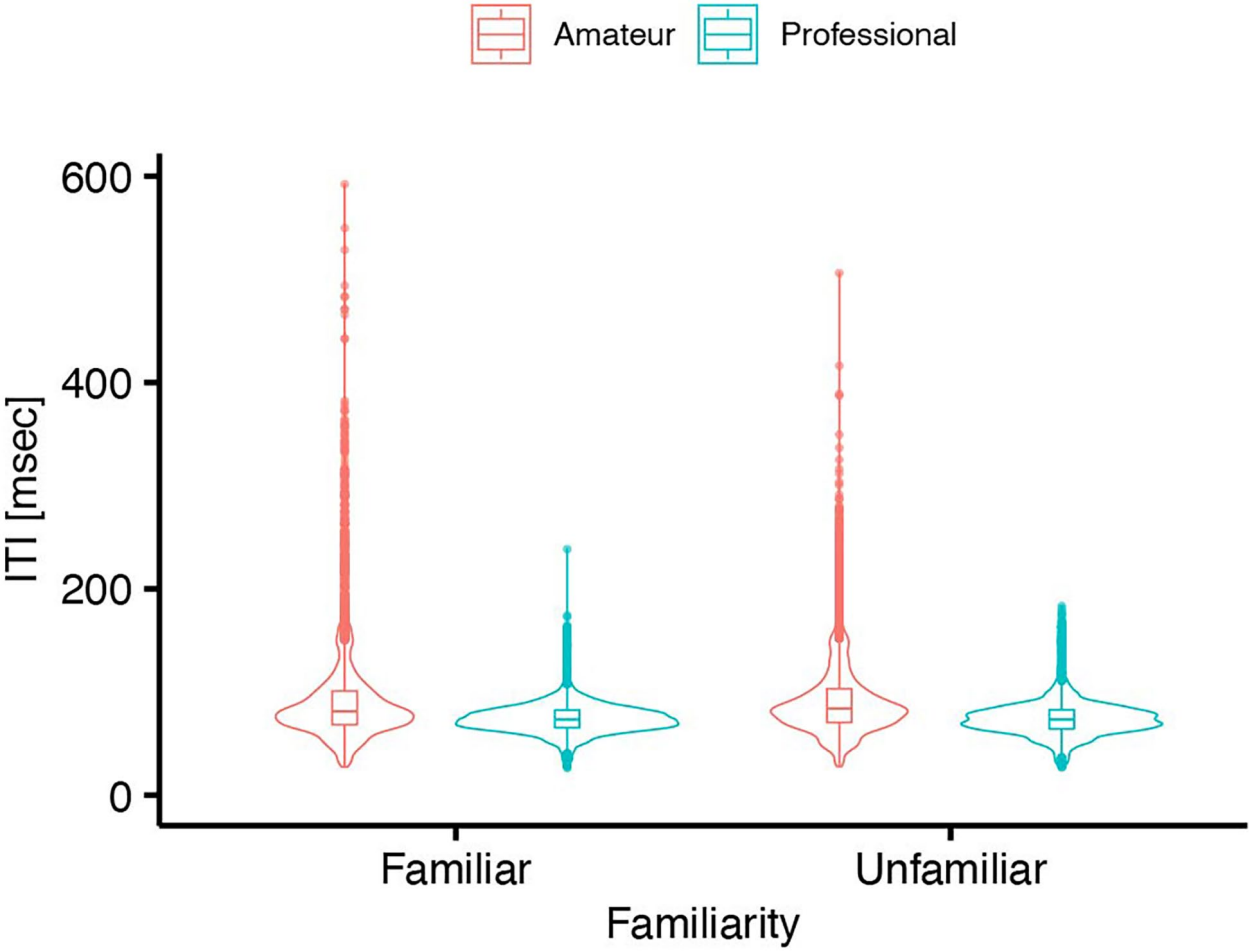
Inter-tap interval of professional and amateur darbuka players under familiar (Li-Lr, Lr-Rr and Li-Rr) and unfamiliar (Ri-Rr, Li-Ri, and Lr-Ri) conditions in the double-finger tapping task (*n* = 8 for the professional group and *n* = 8 for the amateur group).

The LMM revealed no significant interaction between Group and Familiarity in the double-finger tapping task [ $\chi^{2}$ (1) = 0.00054, *p* = 0.98, Figure 5]. The main effect of Familiarity was not significant [ $\chi^{2}$ (1) = 0.71, *p* = 0.40] while that of Group was significant [ $\chi^{2}$ (1) = 5.76, *p* = 0.016, $\eta_{p}^{2}$ = 0.29]. The model showed that the professional players performed the double-finger tapping faster than the amateur players ( β = −0.022, *t* = −2.4, 95% CI = −0.040– −0.0036).

### Variability of ITI and Tapping Amplitude

**Figure 6:**
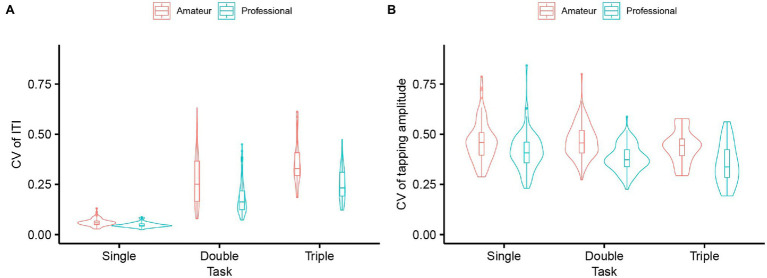
**(A)** Coefficient of variation (CV) of inter-tap interval (ITI) of professional and amateur darbuka players in the single-, double- and triple-finger tapping tasks (*n* = 8 for the professional group and *n* = 8 for the amateur group). **(B)** CV of tapping amplitude of professional and amateur darbuka players in the single-, double- and triple-finger tapping tasks (*n* = 8 for the professional group and *n* = 8 for the amateur group).

As for the CV of ITI, the LMM revealed a significant interaction between Task and Group [ $\chi^{2}$ (2) = 32.30, *p* < 0.01, $\eta_{p}^{2}$ *=* 0.06]. *Post hoc* LMM analyses entering Group as the fixed effect for each task showed the significant main effect of Group in all the tasks [single, $\chi^{2}$ (1) = 7.36, *p* < 0.01, $\eta_{p}^{2}$ = 0.34; double, $\chi^{2}$ (1) = 7.17, *p* < 0.01, $\eta_{p}^{2}$ = 0.34; triple, $\chi^{2}$ (1) = 6.12, *p* < 0.01, $\eta_{p}^{2}$ = 0.39, respectively]. Namely, the professional players showed less variability of ITI than the amateur players in all the tasks [single, β *=* − 0.013, *t* = −2.71, 95% CI = −0.023 – −0.0036; double, β = −0.088, *t* = −2.71, 95% CI = −0.16– −0.023; triple, β = −0.11, *t* = −2.47, 95% CI = −0.21– –0.016, Figure 6A].

As for the CV of tapping amplitude, the LMM revealed a significant interaction between Task and Group [ $\chi^{2}$ (2) = 6.94, *p* < 0.031, $\eta_{p}^{2}$ *=* 0.014]. *Post hoc* LMM analyses entering Group as the fixed effect for each task showed the significant main effect of Group in the single-finger and double-finger tapping tasks [single, $\chi^{2}$ (1) = 4.77, *p* = 0.029, $\eta_{p}^{2}$ = 0.25; double, $\chi^{2}$ (1) = 25.53, *p* < 0.01, $\eta_{p}^{2}$ = 0.63, respectively]. Namely, the professional players showed less variability of tapping amplitude than the amateur players in the single-finger and double-finger tapping tasks (single, β *=* − 0.047, *t* = −2.18, 95% CI = −0.092 – −0.0053; double, β = −0.086, *t* = −4.85, 95% CI = −0.12 – −0.051, Figure 6B). The main effect of Group did not reach a significant level in the triple-finger tapping task but the professional players still showed the tendency of less variability of tapping amplitude [ $\chi^{2}$ (1) = 3.50, *p* = 0.061].

### Correlation Between Tapping Frequency and Musical Experiences

**Figure 7:**
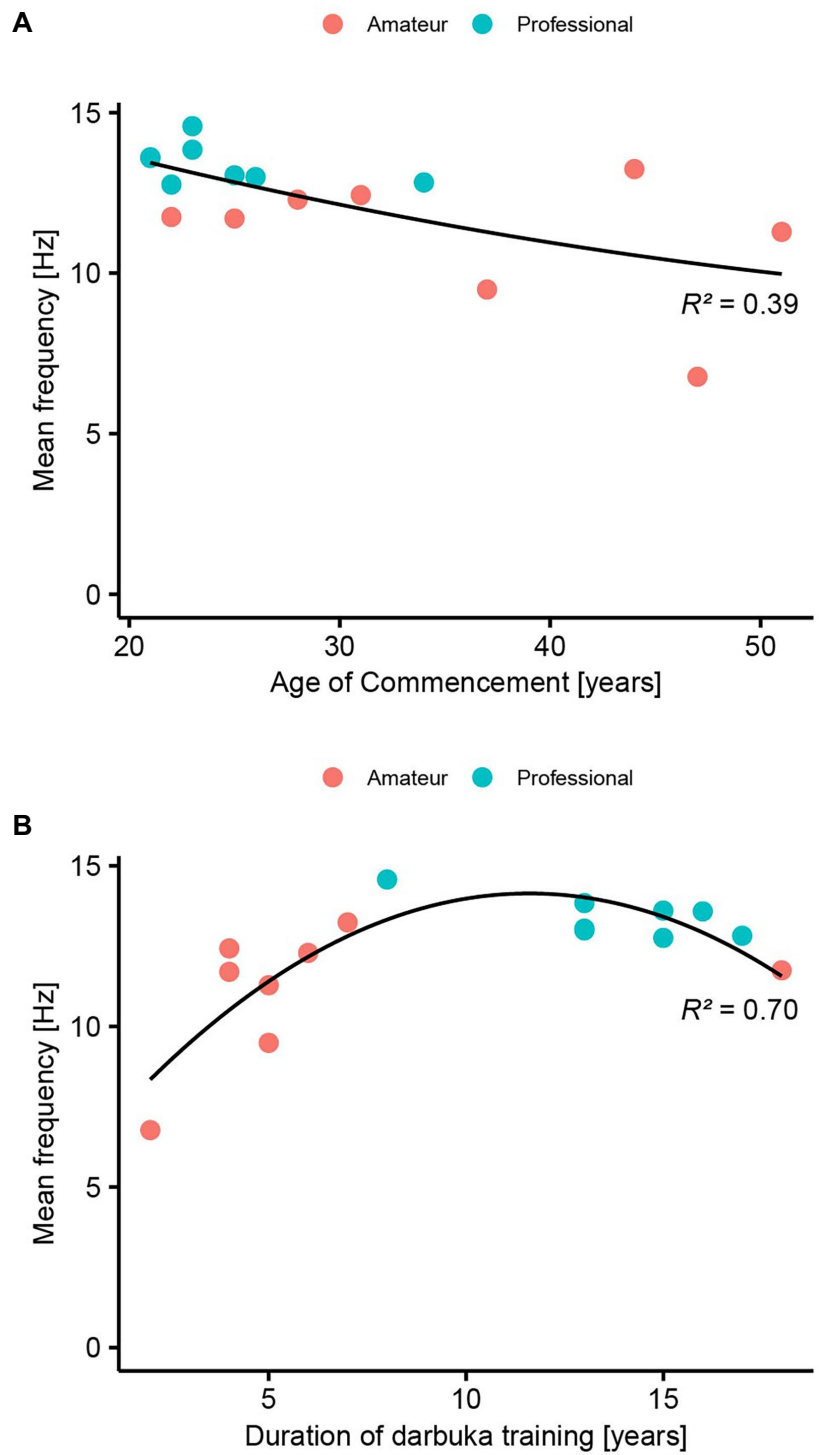
**(A)** Correlation between age at the commencement of darbuka training and tapping frequency in double-finger tapping task. A polynomial fitting curve and the R-squared value are shown. **(B)** Correlation between duration of darbuka training and tapping frequency in double-finger tapping task. A polynomial fitting curve and the *R*-squared value are shown.

Since the professional players tapped faster than the amateur players only in the double-finger tapping task, we calculated the average of tapping frequencies in this task for each participant and correlated it with the age of commencement and duration of darbuka training (Figure 7). The age of commencement of darbuka training was significantly correlated with the average of the tapping frequency in all the participants (Spearman’s *ρ* = −0.58, *p* = 0.020, Figure 7A). The R-square value of the linear fitting model was 0.38 while that of the polynomial fitting model was 0.39. When we subdivided into each of the professional and amateur groups, the correlation did not reach at the significant level (professional, Spearman’s *ρ* = −0.34, *p* = 0.41; amateur, Spearman’s *ρ* = −0.33, *p* = 0.43, respectively).

The duration of darbuka training was significantly correlated with the average of the tapping frequency in all the participants (Spearman’s *ρ* = 0.52, *p* = 0.040, Figure 7B). The R-square value of the linear fitting model was 0.30 while that of the polynomial fitting model was 0.70. When we subdivided into each group, the correlation did not reach a significant level (professional, Spearman’s *ρ* = −0.55, *p* = 0.16; amateur, Spearman’s *ρ* = 0.51, *p* = 0.20, respectively).

In addition, we investigated if the years of age at the time of the experiment related to the tapping frequency. However, there was no significant correlation between the years of age and the average of the tapping frequency (Spearman’s *ρ* = −0.33, *p* = 0.20).

## Discussion

We compared the tapping performance between the professional darbuka players and the matched amateur controls to test the following three hypotheses: (1) Right-handed professional darbuka players would show faster tapping speed than amateur players when they tapped with the familiar fingers to play darbuka (i.e., left-index, left-ring, and right-ring fingers). (2) Professional and amateur darbuka players would show similar tapping-speed asymmetry because both professional and amateur darbuka players practice preferred hand more to play the rhythm patterns in Middle Eastern music. (3) Professional darbuka players would show faster tapping performance than amateur players when they tapped alternately/sequentially with the familiar fingers to play darbuka (left-index, left-ring, and right-ring fingers).

First, we did not find any significant difference in the tapping speed at left-index, left-ring, and right-ring fingers between professional and amateur players in the single-finger tapping task. Thus, the result of this study did not support the first hypothesis. Second, we found that the professional players had less tapping-speed asymmetry than the amateur players. This result did not support the second hypothesis. Third, the professional players showed faster tapping performance in the double-finger tapping task than the amateur players. However, we could not find any significant interaction between the effects of Group and Familiarity in the LMMs in the double-finger tapping task. Thus, the results partially supported the third hypothesis. The professional players showed faster tapping performance when they tapped with not only their familiar fingers but also with unfamiliar fingers.

### The Speed of Single-Finger Tapping

We hypothesized that right-handed skilled professional darbuka players would show faster tapping speed in the single-finger tapping task when they tapped with a left-index finger, a left-ring finger, and a right-ring finger. This was because the darbuka players usually used these fingers for playing the instrument. However, the speed in the single-finger tapping task was not significantly different between the professional and the amateur players. The result is inconsistent with the previous study by Aoki et al. (2005) who found a significant difference in the single-finger tapping speed between the pianists and the non-pianists. We suggest that this inconsistency may be attributed to the following two reasons.

First, the data distribution of musical training background could be wider in the previous study compared to this study. Namely, Aoki et al. (2005) compared the musicians with the non-musicians, while we compared the professional players with amateur players. The amount of experience to play musical instruments was more clearly different in the study by Aoki et al. (2005) compared with this study. Thus, it might be difficult to find the significant difference in the speed between the groups in the single-finger tapping task in this study.

Another reason may be the difference in the joint constraint during the task. In the Aoki et al. (2005) study, the participants placed the palm on a table and were allowed to use only the metacarpophalangeal joint movement but not the other joints (e.g., wrist and elbow joints). On the other hand, the participants in this study were allowed to use upper-arm joints without any constraints to make the task compared with the actual performance. In such unconstrained single-finger tapping, the movements of wrist and elbow joints are involved (Dennerlein et al., 2007). Namely, the participants in this study used not only the finger joint but also the other joint movements (e.g., pronation and supination of the forearm). In this study, the finger was a contact element between hand and the membrane of the drum (i.e., the last element in the chain) and the darbuka players tapped with the fingers in conjunction with the arm rotations and displacements. This was different compared with the previous study by Aoki et al. (2005) in which the wrist and elbow joints were constrained. Thus, unconstrained movements of the upper limb might make it difficult to observe the difference between the professionals and amateurs in the single-finger tapping task in this study.

### Tapping-Speed Asymmetry

Our second hypothesis was that both professional and amateur darbuka players would show similar tapping-speed asymmetry in the single-finger tapping task. This was because both of the professional and amateur darbuka players had used the preferred hand more than the non-preferred hand in their daily performance. Our result showed that both professional and amateur players showed faster performance with the preferred right hand compared with the non-preferred left hand in the single-finger tapping tasks. However, the professional players showed less degree of tapping-speed asymmetry than the amateur players. The result does not support the hypothesis that both professional and amateur darbuka players show a similar degree of tapping-speed asymmetry.

The previous studies showed less tapping-speed asymmetry in musicians compared with non-musicians (Jäncke et al., 1997; Fujii and Oda, 2006). Fujii and Oda (2006) showed that drum-kit players had less degree of tapping-speed asymmetry than non-drummers when they performed the single-hand tapping task with a handheld drumstick. The result of this study was consistent with these previous studies that showed less degree of tapping-speed asymmetry in musicians compared to non-musicians. Drum-kit players have asymmetrical drum setups, such as striking the hi-hat and the snare drum with the right and left hands, respectively. However, in the daily practice of drumming, it is common to use the left and right hands equally, as played in rudiments which are the fundamental drum playing technique. Although the darbuka players use the right hand more than the left hand to play the instrument, motor control of both left and right hands is considered to be important to achieve dexterous musical performance. In addition, the darbuka players usually practice a tremolo technique such as the symmetrical bimanual tapping (Lr–Rr). Taken together, we suggest that acquisition of control skills of both left and right hands may lead to less degree of tapping-speed asymmetry in professional darbuka players.

### Manual Coordination

Our third hypothesis was that the professional darbuka players would show faster speed than the amateur players when they tapped with the left-index, left-ring, and right-ring fingers. This was because the darbuka players use these fingers to play the darbuka. In the double-finger tapping task, our results showed that the professional players performed faster than the amateur players not only when they tapped with the left-index, left-ring, and right-ring fingers (familiar condition), but also when they tapped with the other combinations of fingers (unfamiliar condition).

Why did professional players perform faster tapping in the finger combinations which are not familiar in the usual musical performance? We assume that there might be a transfer or generalization of the motor learning effect from the practiced fingers. In fact, previous studies on finger tapping have shown that the learning effect was transferred from a practiced finger to the other fingers (Schulze et al., 2002; Koeneke et al., 2009; Furuya et al., 2013). For example, Koeneke et al. (2009) made the participants practice tapping 6 days per week for 2 weeks with the left or right middle finger. Interestingly, they found improvements in tapping speed not only in the practiced middle finger but also in the other unpracticed fingers. They assumed that the practice might improve general pacing circuit whose output could be routed to send motor commands to any of the fingers (Koeneke et al., 2009). Thus, although the darbuka players coordinate the left-index, left-ring, and right-ring fingers in their daily practice, the effect might be transferred to the other fingers resulting in the improvement of tapping speed in all the combinations of fingers.

### Variability of Tapping Performance

We found that the professional players showed less variability of ITI and tapping amplitude. While the tapping speed was comparable between the professional and amateur players in the single-finger and triple-finger tasks, the variability was different between the two groups. Moreover, the professional players showed not only faster but also more stable performance compared to the amateur players in the double-finger tapping task. In other words, the professional’s performance was more regular and even in terms of the timing and amplitude when they alternatively tapped with two fingers. The finding was consistent with the previous studies on drummers that showed stable tapping performance in professional players (Fujii and Oda, 2006; Fujii et al., 2010). Producing a regular rhythm and controlling the loudness of sounds are arguably important elements for achieving dexterous musical performance. The results suggest that the professional darbuka players have acquired the stable performance to play the instrument dexterously.

### Age of Commencement and Duration of Darbuka Training

To assess if the intensive practice of darbuka had improved the tapping speed, we analyzed the correlation between the tapping frequency of the double-finger tapping tasks and the experience of the practice of darbuka. As the results, we found a significant negative correlation between the age of commencement and the tapping speed and a positive correlation between the training duration and the tapping speed in the data from all participants. The earlier and the longer they had practiced the darbuka, the faster they showed the tapping performance when alternately coordinating the fingers. The results indicate that the prolonged practice of darbuka from an earlier age of years had improved the manual coordination skill.

We found that the R-squared values of the polynomial fitting models were larger than those of linear fitting models. The polynomial fitting model for the data between the duration of darbuka training and tapping frequency showed inversed U-shaped or ceiling relationship between the two valuables. This suggests more improvement in the early stage of darbuka training than in the later stage. There may be a decay or ceiling effect of tapping speed improvement in the later stage of darbuka training.

The previous study suggested that the tapping skill of musicians was related to the age of commencement of musical practice but not to the duration of musical practice (Jäncke et al., 1997). The previous behavioral and neuroimaging studies also suggest that manual dexterity and the structural brain adaptation in musicians were related to the age of commencement of musical training (Jäncke et al., 1997; Kopiez et al., 2010; Bailey and Penhune, 2012; Baer et al., 2015). These studies suggest that the age of commencement is significant because it influences the developing brain structure and functions. However, it was noteworthy that the age of commencement of musical practice in this study was relatively late compared with the previous studies. The darbuka players in this study started their training over 20 years old while the previous studies emphasize that the age of seven is the critical age to differentiate the course of brain development (Jäncke et al., 1997; Kopiez et al., 2010; Bailey and Penhune, 2012; Baer et al., 2015). Our results suggest that the early commencement of musical training may be important for improving tapping performance even over the age of 20. Nevertheless, we need to interpret the results with caution because there was a significant correlation between the age of commencement and the duration of darbuka training in this study (*r* = −0.62 and *p* = 0.013). For future studies, it would be interesting to control either the age of comment of darbuka training or the duration of darbuka training to investigate how each factor contributes to the improvement of tapping performance. Another limitation of this study was that we could not measure the amount of practice or played hours of the participants. For future studies, it would also be important to assess how the amount of practice or played hours affects the tapping performance of darbuka players.

## Conclusion

The right-handed professional darbuka (hand percussion) players showed significantly faster and more stable tapping performance compared to the amateur players when they tapped with the two fingers as fast and alternate as possible. Interestingly, the professional players showed faster tapping speed in both familiar and unfamiliar patterns of finger coordination. The earlier and the longer they had the darbuka training, the faster they coordinated the fingers. When they tapped with a single finger as fast as possible or tapped with three fingers as fast and even as possible, the speed was similar between the professional and amateur players, but the professionals showed more stable performance. The results suggest that professional darbuka players have acquired fast and stable tapping performance over the prolonged practice.

## Data Availability Statement

The raw data supporting the conclusions of this article will be made available by the authors, without undue reservation.

## Ethics Statement

The studies involving human participants were reviewed and approved by the Ethics Committee of Keio University Shonan Fujisawa Campus. The patients/participants provided their written informed consent to participate in this study. Written informed consent was obtained from the individual(s) for the publication of any potentially identifiable images or data included in this article.

## Author Contributions

KH and SF conceived, designed the study, interpreted the data, and wrote the paper. KH performed the experiment and analyzed the data. All authors contributed to the article and approved the submitted version.

## Funding

This study was supported by Keio Gijuku Academic Development Funds and a Keio SFC Startup Support Grant awarded to SF and the Grants-in-Aid for Scientific Research from the Japan Society for the Promotion of Science and the Ministry of Education Culture, Sports, Science and Technology (20H04092) awarded to SF.

## Conflict of Interest

KH was employed by the company NTT Communication Science Laboratories, NTT Corporation.

The remaining author declares that the research was conducted in the absence of any commercial or financial relationships that could be construed as a potential conflict of interest.

## Publisher’s Note

All claims expressed in this article are solely those of the authors and do not necessarily represent those of their affiliated organizations, or those of the publisher, the editors and the reviewers. Any product that may be evaluated in this article, or claim that may be made by its manufacturer, is not guaranteed or endorsed by the publisher.
