# Supplementary material for: Tapping Performance of Professional and Amateur Darbuka Players
Source: Front Psychol. 2022 Jun 30;13:861821. doi: 10.3389/fpsyg.2022.861821 (PMC9280333; doi:10.3389/fpsyg.2022.861821)
Supplement: Supplementary file 1 [file Data_Sheet_1.DOCX]

Supplementary Material

In this supplementary material, we described the estimated coefficient of linear mixed effect models (LMMs). We conducted the LMMs by using the “lmer” function in the “lme4” package for R (Bates et al. 2015). To evaluate the *p*-values and the 95% confidence interval (CI), we used “lmerTest” package and “confint.merMod” via bootstrapping in “lme4” respectively. The formulas are described following the “lme4” package (see Bates et al. 2015).

# Inter-tap interval (ITI)

## LMM: fixed effects on tapping speed

Formula:

$$ITI \sim Group*Task+\left( 1 | Participant \right)+(1|Trial)$$

**Supplementary Table 1.** Fixed effects of Group * Task on the tapping speed in the single-, double- and triple-finger tapping tasks by the darbuka players.

|  | Estimate | Std. Error | Df | *t* value | *p*-value | CI 2.50% | CI 97.50% |
| --- | --- | --- | --- | --- | --- | --- | --- |
| (Intercept) | 0.144294 | 0.006248 | 14.382 | 23.09502 | < 0.01 | 0.132251 | 0.156554 |
| Group [Professional] | -0.00744 | 0.008783 | 14.05399 | -0.84658 | 0.411415 | -0.02554 | 0.010124 |
| Task [Double] | -0.04772 | 0.00043 | 47586.61 | -110.993 | < 0.01 | -0.04858 | -0.04692 |
| Task [Triple] | -0.0521 | 0.000636 | 47586.69 | -81.9761 | < 0.01 | -0.05333 | -0.05082 |
| Group [Professional]: Task [Double] | -0.01425 | 0.000582 | 47586.44 | -24.4996 | < 0.01 | -0.01536 | -0.01307 |
| Group [Professional]: Task [Triple] | -0.02322 | 0.000844 | 47586.76 | -27.5132 | < 0.01 | -0.02502 | -0.0217 |

### Separate post-hoc LMM: fixed effects on tapping speed of single-finger tapping task

Formula:

$$ITI \sim Group+\left( 1 | Participant \right)+(1|Trial)$$

**Supplementary Table 2.** Fixed effects of Group on the tapping speed of the single-finger tapping task by the darbuka players.

|  | Estimate | Std. Error | Df | *t* value | *p*-value | CI 2.50% | CI 97.50% |
| --- | --- | --- | --- | --- | --- | --- | --- |
| (Intercept) | 0.143514 | 0.004249 | 14.25999 | 33.77874 | < 0.01 | 0.135244 | 0.152792 |
| Group [Professional] | -0.00657 | 0.005979 | 13.99359 | -1.09963 | 0.29005 | -0.01826 | 0.005706 |

### Separate post-hoc LMM: fixed effects on tapping speed of double-finger tapping task

Formula:

$$ITI \sim Group+\left( 1 | Participant \right)+\left( 1 | Trial \right)$$

**Supplementary Table 3.** Fixed effects of Group on the tapping speed of the double-finger tapping task by the darbuka players.

|  | Estimate | Std. Error | Df | *t* value | *p*-value | CI 2.50% | CI 97.50% |
| --- | --- | --- | --- | --- | --- | --- | --- |
| (Intercept) | 0.096684 | 0.006481 | 14.58131 | 14.91764 | < 0.01 | 0.083538 | 0.108878 |
| Group [Professional] | -0.02176 | 0.00906 | 13.97645 | -2.40205 | 0.030774 | -0.03944 | -0.00311 |

### Separate post-hoc LMM: fixed effects on tapping speed of triple-finger tapping task

Formula:

$$ITI \sim Group+\left( 1 | Participant \right)+\left( 1 | Trial \right)$$

**Supplementary Table 4.** Fixed effects of Group on the tapping speed of the triple-finger tapping task by the darbuka players.

|  | Estimate | Std. Error | Df | *t* value | *p*-value | CI 2.50% | CI 97.50% |
| --- | --- | --- | --- | --- | --- | --- | --- |
| (Intercept) | 0.101672 | 0.015205 | 13.97278 | 6.686736 | < 0.01 | 0.07409 | 0.128791 |
| Group [Professional] | -0.03798 | 0.021491 | 13.94073 | -1.76733 | 0.099045 | -0.08078 | 0.005987 |

## LMM: fixed effects on single-finger tapping

Formula:

$$ITI \sim Group*Hand*Finger+\left( 1 | Participant \right)+\left( 1 | Trial \right)$$

**Supplementary Table 5.** Fixed effects of Group*Hand*Finger on the tapping speed in the single-finger tapping task by the darbuka players.

|  | Estimate | Std. Error | df | *t* value | *p*-value | CI 2.50% | CI 97.50% |
| --- | --- | --- | --- | --- | --- | --- | --- |
| (Intercept) | 0.140955 | 0.004368 | 14.32588 | 32.27129 | < 0.01 | 0.132253 | 0.148802 |
| Group [Professional] | -0.00806 | 0.006148 | 14.06519 | -1.3103 | 0.21109 | -0.01954 | 0.003906 |
| Hand [Left] | 0.011091 | 0.000419 | 11595.93 | 26.47598 | < 0.01 | 0.010302 | 0.01195 |
| Finger [Ring] | -0.00657 | 0.000366 | 11595.34 | -17.9206 | < 0.01 | -0.00733 | -0.00588 |
| Group [Professional]: Hand [Left] | -0.00252 | 0.000555 | 11595.54 | -4.54501 | < 0.01 | -0.0036 | -0.00147 |
| Group [Professional]: Finger [Left] | 0.006674 | 0.000506 | 11595.27 | 13.18142 | < 0.01 | 0.005714 | 0.007713 |
| Hand [Left]: Finger [Ring] | 0.005439 | 0.000561 | 11595.34 | 9.703321 | < 0.01 | 0.004438 | 0.006486 |
| Group [Professional]: Hand [Left]:  Finger [Ring] | -0.00562 | 0.000758 | 11595.19 | -7.42423 | < 0.01 | -0.00704 | -0.00409 |

### Separate post-hoc LMM: fixed effects on tapping speed of single-finger tapping

Formula

$$ITI \sim Group*Familiarity+\left( 1 | Participant \right)+(1|Trial)$$

**Supplementary Table 6.** Fixed effects of Group*Familiarity on the tapping speed in the single-finger tapping task by the darbuka players.

|  | Estimate | Std. Error | df | *t* value | *p*-value | CI 2.50% | CI 97.50% |
| --- | --- | --- | --- | --- | --- | --- | --- |
| (Intercept) | 0.096896 | 0.006488 | 14.62603 | 14.93402 | < 0.01 | 0.083489 | 0.109505 |
| Group [Professional] | -0.02176 | 0.009069 | 14.01562 | -2.39975 | 0.030866 | -0.04044 | -0.00357 |
| Familiarity [Unfamiliar] | -0.00042 | 0.000504 | 29825.3 | -0.84149 | 0.40008 | -0.0015 | 0.000568 |
| Group [Professional]:  Familiarity [Unfamiliar] | 1.55E-05 | 0.000671 | 29825.37 | 0.023154 | 0.981528 | -0.00127 | 0.001451 |

#### Separate post-hoc LMM: fixed effects on the tapping speed of familiar single-finger tapping

Formula:

$$ITI \sim Group+\left( 1 | Participant \right)+\left( 1 | Trial \right)$$

**Supplementary Table 7.** Fixed effects of Group on the tapping speed of the familiar single-finger tapping task by the darbuka players.

|  | Estimate | Std. Error | df | *t* value | *p*-value | CI 2.50% | CI 97.50% |
| --- | --- | --- | --- | --- | --- | --- | --- |
| (Intercept) | 0.144978 | 0.00481 | 14.11394 | 30.14383 | < 0.01 | 0.135374 | 0.154725 |
| Group [Professional] | -0.00663 | 0.006786 | 13.98989 | -0.97728 | 0.345028 | -0.02029 | 0.006372 |

#### Separate post-hoc LMM: fixed effects on tapping speed of unfamiliar single-finger tapping

Formula:

$$ITI \sim Group+\left( 1 | Participant \right)+\left( 1 | Trial \right)$$

**Supplementary Table 8.** Fixed effects of Group on the tapping speed of the unfamiliar single-finger tapping task by the darbuka players.

|  | Estimate | Std. Error | df | *t* value | *p*-value | CI 2.50% | CI 97.50% |
| --- | --- | --- | --- | --- | --- | --- | --- |
| (Intercept) | 0.141183 | 0.003562 | 14.67402 | 39.63286 | < 0.01 | 0.133959 | 0.147594 |
| Group [Professional] | -0.00811 | 0.004972 | 13.99768 | -1.63163 | 0.125043 | -0.01824 | 0.001183 |

### Separate post-hoc LMM: fixed effects on tapping speed of single-finger tapping of professional players

Formula:

$$ITI \sim Hand+\left( 1 | Participant \right)+\left( 1 | Trial \right)$$

**Supplementary Table 9.** Fixed effects of Hand on the tapping speed in the single-finger tapping task by the professional darbuka players.

|  | Estimate | Std. Error | df | *t* value | *p*-value | CI 2.50% | CI 97.50% |
| --- | --- | --- | --- | --- | --- | --- | --- |
| (Intercept) | 0.132959 | 0.001809 | 8.287183 | 73.50395 | < 0.01 | 0.129381 | 0.136643 |
| Hand [Left] | 0.008473 | 0.000212 | 6240.081 | 39.97699 | < 0.01 | 0.008053 | 0.00887 |

### Separate post-hoc LMM: fixed effects on tapping speed of single-finger tapping of amateur players

Formula:

$$ITI \sim Hand+\left( 1 | Participant \right)+\left( 1 | Trial \right)$$

**Supplementary Table 10.** Fixed effects of Hand on the tapping speed in the single-finger tapping task by the amateur darbuka players.

|  | Estimate | Std. Error | df | *t* value | *p*-value | CI 2.50% | CI 97.50% |
| --- | --- | --- | --- | --- | --- | --- | --- |
| (Intercept) | 0.137649 | 0.005923 | 7.02832 | 23.23953 | < 0.01 | 0.125566 | 0.148311 |
| Hand [Left] | 0.013741 | 0.000333 | 5358.549 | 41.31015 | < 0.01 | 0.01309 | 0.01442 |

## Separate post-hoc LMM: fixed effects on tapping speed of double-finger tapping

Formula:

$$ITI \sim Group*Familiarity+\left( 1 | Participant \right)+(1|Trial)$$

**Supplementary Table 11:** Fixed effects of Group*Familiarity on the tapping speed in the double-finger tapping task by the darbuka players.

|  | Estimate | Std. Error | df | *t* value | *p*-value | CI 2.50% | CI 97.50% |
| --- | --- | --- | --- | --- | --- | --- | --- |
| (Intercept) | 0.096896 | 0.006488 | 14.62603 | 14.93402 | < 0.01 | 0.083489 | 0.109505 |
| Group [Professional] | -0.02176 | 0.009069 | 14.01562 | -2.39975 | 0.030866 | -0.04044 | -0.00357 |
| Familiarity [Unfamiliar] | -0.00042 | 0.000504 | 29825.3 | -0.84149 | 0.40008 | -0.0015 | 0.000568 |
| Group [Professional]:  Familiarity [Unfamiliar] | 1.55E-05 | 0.000671 | 29825.37 | 0.023154 | 0.981528 | -0.00127 | 0.001451 |

# Coefficient of variance (CV) of ITI

## LMM: fixed effects on CV of ITI

Formula:

$$CV of ITI \sim Group*Task+\left( 1 | Participant \right)+(1|Trial)$$

**Supplementary Table 12.** Fixed effects of Group * Task on the CV of ITI in the single-, double- and triple-finger tapping tasks by the darbuka players.

|  | Estimate | Std. Error | df | *t* value | *p*-value | CI 2.50% | CI 97.50% |
| --- | --- | --- | --- | --- | --- | --- | --- |
| (Intercept) | 0.063842 | 0.017611 | 18.84157 | 3.625227 | 0.001822 | 0.028155 | 0.097157 |
| Group [Professional] | -0.01588 | 0.023324 | 18.62693 | -0.68088 | 0.504322 | -0.0563 | 0.028052 |
| Task [Double] | 0.205333 | 0.00985 | 500.0725 | 20.8457 | < 0.01 | 0.187389 | 0.225197 |
| Task [Triple] | 0.296086 | 0.016944 | 499.9658 | 17.47443 | < 0.01 | 0.264074 | 0.328539 |
| Group [Professional]: Task [Double] | -0.07206 | 0.01389 | 500.0276 | -5.18762 | < 0.01 | -0.0992 | -0.04623 |
| Group [Professional]: Task [Triple] | -0.09536 | 0.023921 | 499.9386 | -3.98669 | < 0.01 | -0.14163 | -0.0518 |

### Separate post-hoc LMM: fixed effects on CV of ITI of single-finger tapping task

Formula:

$$CV of ITI \sim Group+\left( 1 | Participant \right)+(1|Trial)$$

**Supplementary Table 13.** Fixed effects of Group on the CV of ITI in the single-finger tapping tasks by the darbuka players.

|  | Estimate | Std. Error | df | *t* value | *p*-value | CI 2.50% | CI 97.50% |
| --- | --- | --- | --- | --- | --- | --- | --- |
| (Intercept) | 0.060531 | 0.003466 | 13.99866 | 17.46325 | < 0.01 | 0.053603 | 0.067702 |
| Group [Professional] | -0.01257 | 0.004634 | 14.07719 | -2.71278 | 0.016765 | -0.02271 | -0.00357 |

### Separate post-hoc LMM: fixed effects on CV of ITI of double-finger tapping task

Formula:

$$CV of ITI \sim Group+\left( 1 | Participant \right)+(1|Trial)$$

**Supplementary Table 14.** Fixed effects of Group on the CV of ITI in the double-finger tapping tasks by the darbuka players.

|  | Estimate | Std. Error | df | *t* value | *p*-value | CI 2.50% | CI 97.50% |
| --- | --- | --- | --- | --- | --- | --- | --- |
| (Intercept) | 0.269175 | 0.025104 | 14.35916 | 10.72257 | < 0.01 | 0.219487 | 0.319121 |
| Group [Professional] | -0.08786 | 0.03281 | 14.03174 | -2.6777 | 0.017999 | -0.15916 | -0.02314 |

### Separate post-hoc LMM: fixed effects on CV of ITI of triple-finger tapping task

Formula:

$$CV of ITI \sim Group+\left( 1 | Participant \right)+(1|Trial)$$

**Supplementary Table 15.** Fixed effects of Group on the CV of ITI in the triple-finger tapping tasks by the darbuka players.

|  | Estimate | Std. Error | df | *t* value | *p*-value | CI 2.50% | CI 97.50% |
| --- | --- | --- | --- | --- | --- | --- | --- |
| (Intercept) | 0.359928 | 0.031808 | 13.99999 | 11.31581 | < 0.01 | 0.294515 | 0.419938 |
| Group [Professional] | -0.11125 | 0.044983 | 13.99999 | -2.47309 | 0.026824 | -0.20539 | -0.01553 |

# Coefficient of variance of tapping amplitude

## LMM: fixed effects on CV of tapping amplitude

Formula:

$$CV of tapping amplitude \sim Group*Task+\left( 1 | Participant \right)+(1|Trial)$$

**Supplementary Table 16.** Fixed effects of Group * Task on the CV of tapping amplitude in the single-, double- and triple-finger tapping tasks by the darbuka players.

|  | Estimate | Std. Error | df | *t* value | *p*-value | CI 2.50% | CI 97.50% |
| --- | --- | --- | --- | --- | --- | --- | --- |
| (Intercept) | 0.46229 | 0.01447 | 22.17464 | 31.94866 | < 0.01 | 0.435014 | 0.489938 |
| Group [Professional] | -0.04771 | 0.020408 | 21.94297 | -2.33781 | 0.028922 | -0.08696 | -0.01002 |
| Task [Double] | 0.003787 | 0.010499 | 502.2583 | 0.360682 | 0.718489 | -0.01472 | 0.02578 |
| Task [Triple] | -0.02493 | 0.018061 | 502.0889 | -1.38039 | 0.168081 | -0.05873 | 0.011355 |
| Group [Professional]: Task [Double] | -0.038 | 0.014806 | 502.1871 | -2.56656 | 0.01056 | -0.06854 | -0.00868 |
| Group [Professional]: Task [Triple] | -0.03702 | 0.025498 | 502.0456 | -1.45189 | 0.147155 | -0.09526 | 0.012249 |

### Separate post-hoc LMM: fixed effects on CV of tapping amplitude of single-finger tapping task

Formula:

$$CV of tapping amplitude \sim Group+\left( 1 | Participant \right)+(1|Trial)$$

**Supplementary Table 17.** Fixed effects of Group on the CV of tapping amplitude in the single-finger tapping tasks by the darbuka players.

|  | Estimate | Std. Error | df | *t* value | *p*-value | CI 2.50% | CI 97.50% |
| --- | --- | --- | --- | --- | --- | --- | --- |
| (Intercept) | 0.46163 | 0.015292 | 14.35048 | 30.18792 | < 0.01 | 0.431101 | 0.491933 |
| Group [Professional] | -0.04705 | 0.02155 | 14.16948 | -2.18339 | 0.046306 | -0.09198 | -0.00525 |

### Separate post-hoc LMM: fixed effects on CV of tapping amplitude of double-finger tapping task

Formula:

$$CV of tapping amplitude \sim Group+\left( 1 | Participant \right)+(1|Trial)$$

**Supplementary Table 18.** Fixed effects of Group on the CV of tapping amplitude in the double-finger tapping tasks by the darbuka players.

|  | Estimate | Std. Error | df | *t* value | *p*-value | CI 2.50% | CI 97.50% |
| --- | --- | --- | --- | --- | --- | --- | --- |
| (Intercept) | 0.466076 | 0.012528 | 13.96587 | 37.20336 | < 0.01 | 0.440513 | 0.490834 |
| Group [Professional] | -0.08605 | 0.017739 | 14.03273 | -4.85073 | < 0.01 | -0.12223 | -0.05086 |

### Separate post-hoc LMM: fixed effects on CV of tapping amplitude of triple-finger tapping task

Formula:

$$CV of tapping amplitude \sim Group+\left( 1 | Participant \right)+(1|Trial)$$

**Supplementary Table 19.** Fixed effects of Group on the CV of tapping amplitude in the triple-finger tapping tasks by the darbuka players.

|  | Estimate | Std. Error | df | *t* value | *p*-value | CI 2.50% | CI 97.50% |
| --- | --- | --- | --- | --- | --- | --- | --- |
| (Intercept) | 0.437358 | 0.032014 | 14 | 13.66155 | < 0.01 | 0.3674 | 0.494733 |
| Group [Professional] | -0.08473 | 0.045274 | 14 | -1.87151 | 0.082318 | -0.17708 | 0.007822 |

# References

Bates, Douglas, Martin Mächler, Ben Bolker, and Steve Walker. 2015. “Fitting Linear Mixed-Effects Models Using Lme4.” *Journal of Statistical Software* 67 (October): 1–48.
